# Cardiopulmonary Exercise Testing (CPET) Guided Sub‐Anaerobic Threshold Rehabilitation in MELAS Syndrome: A Case Report

**DOI:** 10.1002/kjm2.70266

**Published:** 2026-07-15

**Authors:** Yu‐Lien Tsai, Kun‐Yuan Cheng, Yen‐Sen Lu, Ko‐Long Lin

**Affiliations:** ^1^ Department of Post‐Baccalaureate Medicine, College of Medicine Kaohsiung Medical University Kaohsiung Taiwan; ^2^ Department of Physical Medicine and Rehabilitation, Kaohsiung Medical University Hospital Kaohsiung Medical University Kaohsiung Taiwan; ^3^ Department of Physical Medicine and Rehabilitation, Faculty of Medicine, College of Medicine Kaohsiung Medical University Kaohsiung Taiwan

1

MELAS (mitochondrial encephalomyopathy, lactic acidosis, and stroke‐like episodes) is a progressive mitochondrial disorder causing ATP deficiency and lactic acidosis. Stroke‐like episodes (SLEs) are driven by neuronal hyperexcitability, defined by consensus as a subacute, evolving brain syndrome driven by seizure activity. Baseline serum lactate is significantly associated with SLE risk in m.3243A>G patients [1]. While a direct causal link between exceeding anaerobic threshold (AT) and triggering SLEs has not been established, aerobic training has been shown to be safe in patients with mtDNA mutations [2], and minimizing systemic metabolic stress through below AT training represents a prudent approach to reduce potential metabolic triggers.

A 49‐year‐old man with type 2 diabetes, hyperlipidemia, and MELAS syndrome (MT‐TL1 m.3243A>G) triggered by metformin in 2019, presented with acute left‐sided weakness following resistance exercise with elevated serum lactate. Neuroimaging confirmed acute ischemic stroke in the right ventral medulla with right V4 vertebral artery occlusion, distinct from prior metabolic lesions. Examination revealed bilateral sensorineural hearing loss and left lower‐extremity weakness (Brunnstrom stage III) with preserved cognition. After stable condition, he received an inpatient rehabilitation program.

A symptom‐limited CPET was performed using a cycle ergometer (ramp protocol: 10 W/min) with breath‐by‐breath gas analysis. AT was determined using V̇E/V̇O_2_ and V̇E/V̇CO_2_ methods; maximal effort was confirmed by RER > 1.1.

To our knowledge, this is the first reported case utilizing CPET‐derived AT to guide exercise prescription in MELAS‐associated stroke. While aerobic training benefits have been demonstrated in mitochondrial myopathy, no prior study has applied AT‐targeted rehabilitation in the MELAS population.

We prescribed an exercise‐training program according to his CPET results in Table 1.

**TABLE 1:** Clinical and cardiopulmonary exercise testing outcomes before and after 12 weeks of CPET‐guided sub‐anaerobic threshold rehabilitation.

Variables	Before	After
Barthel index	55	75
Serum lactate (m mol/L)	5.15	2.93
Anaerobic threshold (AT)		
Workload (Watts)	22	25
VE (liter/min)	24.2	28.7
VO_2_ (mL/min/kg)	11.61	13.6
HR (bpm)	140	140
Peak exercise		
Workload (Watts)	40	55
METs	4.5	4.63
VE (liter/min)	35.3	37.8
VO_2_ (mL/kg/min)	15.78	16.23
RER	1.10	1.17
HR (bpm)	155	153

A 12‐week program was implemented with exercise intensity strictly capped below AT (140 bpm). Real‐time heart rate monitoring ensured the patient remained within the safe aerobic window. The intervention consisted of 30‐min cycle ergometer sessions five times a week.

As shown in Table 1, improved work capacity with unchanged maximal oxygen uptake is consistent with enhanced mitochondrial efficiency and biogenesis rather than increased maximal oxidative capacity, which reflects the absolute physiological ceiling imposed by mitochondrial DNA mutations [2, 3]. The increase in AT workload substantially expands the patient's aerobic buffer, enhancing his ability to sustain submaximal activity and improving daily function and quality of life, as evidenced by a rise in Barthel Index scores. These findings align with prior evidence that aerobic training improves oxidative capacity and is safe in mtDNA mutation patients [2]. Resting serum lactate progressively declined, consistent with prior findings (the lactate reduction is consistent with studies) demonstrating approximately 50% decreases in resting venous lactate after 8 weeks of aerobic training in mitochondrial myopathy [4, 5]. No SLEs or adverse events occurred during training.

This case demonstrates that CPET is a valuable tool for all the patients with mitochondrial myopathy. By utilizing AT to define a metabolic safety window, clinicians can prescribe precise heart rate‐guided exercise that improves submaximal work capacity, reduces resting lactate, and enhances daily function [2]. We propose CPET‐guided exercise prescription as part of standard care for patients with impaired aerobic oxidative metabolism.

## Conflicts of Interest

All authors take responsibility for all aspects of the reliability and freedom from bias of the data presented and their discussed interpretation.

## Data Availability

The data that support the findings of this study are available from the corresponding author upon reasonable request.
